# Tomato *DCL2b* is required for the biosynthesis of 22-nt small RNAs, the resulting secondary siRNAs, and the host defense against ToMV

**DOI:** 10.1038/s41438-018-0073-7

**Published:** 2018-09-01

**Authors:** Tian Wang, Zhiqi Deng, Xi Zhang, Hongzheng Wang, Yu Wang, Xiuying Liu, Songyu Liu, Feng Xu, Tao Li, Daqi Fu, Benzhong Zhu, Yunbo Luo, Hongliang Zhu

**Affiliations:** 1 College of Food Science and Nutritional Engineering, China Agricultural University, 100083 Beijing, China; 2 Key Laboratory of Horticultural Plant Biology, Ministry of Education, College of Horticulture and Forestry Sciences, Huazhong Agricultural University, 430070 Wuhan, China; 3 State Key Laboratory of Protein and Plant Gene Research, College of Life Sciences, Peking University, 100871 Beijing, China; 4 State Key Laboratory of Plant Genomics, Institute of Genetics and Developmental Biology, Chinese Academy of Sciences, 100101 Beijing, China; 5 State Key Laboratory of Agro-Biotechnology, College of Biological Sciences, China Agricultural University, 100193 Beijing, China; 6 National Maize Improvement Center, China Agricultural University, 100193 Beijing, China; 7 Institute of Environment and Sustainable Development in Agriculture, Chinese Academy of Agricultural Sciences, 100081 Beijing, China

## Abstract

The tomato encode four functional *DCL* families, of which *DCL2* is poorly studied. Here, we generated loss-of-function mutants for a tomato *DCL2* gene, *dcl2b*, and we identified its major role in defending against tomato mosaic virus in relation to both natural and manual infections. Genome-wide small RNA expression profiling revealed that *DCL2b* was required for the processing 22-nt small RNAs, including a few species of miRNAs. Interestingly, these *DCL2b*-dependent 22-nt miRNAs functioned similarly to the *DCL1*-produced 22-nt miRNAs in *Arabidopsis* and could serve as triggers to generate a class of secondary siRNAs. In particular, the majority of secondary siRNAs were derived from plant defense genes when the plants were challenged with viruses. We also examined differentially expressed genes in *dcl2b* through RNA-seq and observed that numerous genes were associated with mitochondrial metabolism and hormone signaling under virus-free conditions. Notably, when the loss-of-function *dcl2b* mutant was challenged with tomato mosaic virus, a group of defense response genes was activated, whereas the genes related to lipid metabolism were suppressed. Together, our findings provided new insights into the roles of tomato *DCL2b* in small RNA biogenesis and in antiviral defense.

## Short sequences provide pathogen protection

A protein that processes gene-regulating RNA molecules helps defend tomato plants against viral infections. The various *DCL* proteins play a prominent role in processing tiny RNAs known as microRNA and short interfering RNA, which in turn modulate gene activity. Researchers led by Hongliang Zhu at China Agricultural University set out to study a *DCL* protein produced by tomatoes that remains poorly defined from a functional perspective. The researchers did not notice any prominent abnormalities when they genetically manipulated plants to eliminate expression of the *DCL2b* gene. However, these *DLC2b*-deficient plants proved disproportionately vulnerable to tomato mosaic virus, with infection producing striking developmental defects. Zhu and colleagues determined that loss of this gene disrupts processing of a subset of regulatory RNAs that may help mediate the plant antiviral response.

## Introduction

RNA silencing plays key roles in regulating endogenous gene expression, suppressing transposon activity, silencing transgenes, responding to environmental stimuli and combatting viral infection^1,2^. Small RNAs (sRNAs), including miRNA and siRNA, are loaded into an Argonaute (AGO) effector protein to form RNA-induced silencing complexes to repress complementary target RNA at a posttranscriptional gene silencing (PTGS) level through cleavage and/or the inhibition of translation. RNA-induced silencing complexes can also repress gene expression at a transcriptional gene (TGS) silencing level^3,4^.

sRNAs are generated by *DCL* proteins. In *Arabidopsis*, four *DCL* family proteins have been found to cleave stem-loop or double-stranded (ds) RNA precursors into miRNAs or siRNAs of specific sizes^5^. *DCL1* primarily processes hairpin RNA into the 21-nt miRNA that is involved in PTGS^6,7^. However, *DCL1* can also produce 22-nt miRNAs from bulged precursors that in turn lead to the production of secondary siRNAs. *DCL2* is required for the biogenesis of 22-nt siRNAs from endogenous inverted repeat loci^8,9^. *DCL3* produces TGS-engaged 24-nt siRNAs from RNA-dependent RNA Polymerase 2 (RDR2)-dependent dsRNAs^4,10^. *DCL4* produces 21-nt siRNAs from RDR6-dependent dsRNA in the PTGS pathway^11,12^. In addition, *DCL3* and *DCL2* could also generate secondary siRNA (sec-siRNAs) from RDR6-dependent dsRNA in a *dcl4* mutant^13^.

Sec-siRNAs typically exhibit a phased pattern, and they have recently been shown to be derived from a large set of loci; they play important roles in plant development and disease resistance^14,15^. Normally, sec-siRNAs are processed from 3′ cleaved transcripts that are targeted by *DCL1*-generated 22-nt miRNA and trans-acting siRNA (tasiRNA)^16,17^, except for 21-nt miR390^18^. The 3′ cleaved transcripts are amplified into dsRNAs by RDR6 and then cleaved by *DCL4* into 21-nt ‘head-to-tail’-phased, sec-siRNAs^14,18^ or cleaved by *DCL3* into 24-nt sec-siRNA^12^. These sec-siRNAs are called phased siRNA (phasiRNA) if they are generated from coding transcripts, but they are known as tasiRNA if they come from noncoding transcripts. Many miRNAs could trigger the production of sec-siRNAs from various RNA transcripts, such as the pairs miR173 and *TAS1/2*, miR390 and *TAS3*, miR393 and *TIR/AFB*, miR7122 and *PPR*, and miR482 and *NBS-LRR*^14,15^.

Over the past few decades, the biological roles of *DCL1*, *DCL3* and *DCL4* have been well studied, and the *DCL2* was considered as a substitute for *DCL4* for defense against viruses^19,20^. Importantly, *DCL2* plays a primary role in transgene silencing, especially in the sense transgene-induced silencing and transitivity of hairpin-induced transgene silencing^21,22^. *DCL2* is also engaged in plant development and systemic RNA silencing, and *DCL4* attenuates systemic PTGS^8,23,24^. When the *DCL4* function is impaired, endogenous genes (*SMXL4* and *SMXL5*) are excessively silenced by 22-nt siRNA that are produced from the *DCL2*- and *RDR6*-dependent transitive PTGS pathway. In wild type (WT) plants, *DCL4* outcompetes *DCL2* for the same dsRNA templates, which prevents or limits the deleterious effects of this endogenous silencing by *DCL2*^8^. *DCL2* can enhance the recruitment of RDR6 to target transcripts and the production of sec-siRNAs. The *DCL2*-produced 22-nt siRNA can also trigger the production of 21-nt sec-siRNA from target mRNA^9,23,24^. This type of siRNA amplification is essential for enhancing the PTGS efficiency. It is now proposed that there is a dual-defense strategy for plant viruses to break. *DCL4* is the primary defender that attacks viruses in initially infected cells through virus-induced gene silencing in autonomous cells. Once the *DCL4* activity is inhibited by the viral suppressors of RNA silencing, *DCL2* and *DCL2*-processed/dependent siRNAs are required to trigger non-cell autonomous (virus-induced gene silencing) and protect the recipient cells from further invasion by plant viruses^23,24^.

The tomato (*Solanum lycopersicum*) is the seventh-most important crop species and the second-most consumed vegetable in the world^25^. An analysis of tomato *DCL1* and *DCL3*-silencing mutants indicates that *DCL1* produces canonical miRNAs and a few 21-nt siRNAs^7^, and *DCL3* is involved in the biosynthesis of heterochromatic 24-nt siRNAs and long miRNAs^10^. *DCL4* is required for the production of 21-nt tasiRNAs that in turn target the *ARFs* to alter tomato leaf development^11^. However, the functions of tomato *DCL2* remain unknown. Previously, we cloned the full-length cDNA sequences of four tomato *DCL2* subfamily members, *DCL2a*, *DCL2b*, *DCL2c*, and *DCL2d* for expression pattern analysis, and we showed that the *DCL2b* expression is much higher than that of other *DCL2s*, implying its predominant role in biology^26^.

Here, we generated loss-of-function *dcl2b* mutants using the CRISPR/Cas9 genome-editing system. The *dcl2b* mutants did not show developmental defects under normal conditions. When infected by tomato mosaic virus (ToMV), however, the *dcl2b* mutants displayed more severe developmental defects consisting of strange narrow patterns on the leaves, flowers and fruits compared to the WT. Even *DCL4* was still functional, indicating that *DCL2b* played a major role in the defense against ToMV. We performed genome-wide sRNA expression profiling and found that *DCL2b* was required for the biogenesis of 22-nt sRNA, including some 22-nt miRNAs that would otherwise be produced by *DCL1* in *Arabidopsis*, leading to sec-siRNA production. The RNA-seq analysis showed that numerous genes associated with mitochondrial metabolism and hormone signaling changed significantly under virus-free conditions. When the ToMV engaged in infection, the tomato plants activated the genes involved in a response pathway to the stimulus and suppressed a lipid metabolism pathway. Collectively, our results demonstrated that tomato *DCL2b* played a critical role in antiviral defense by regulating the biogenesis of a group of 22-nt miRNAs and subsequently sec-siRNAs.

## Results

### Generation of *dcl2b* mutants using the CRISPR/Cas9 gene-editing system

To generate null alleles of loss-of-function mutants, we used the CRISPR/Cas9 gene-editing system to knock out *DCL2b* in tomatoes. Four genomic sites were targeted for cleavage (Figure S1a), and then transgenic plants were genotyped through the direct sequencing of PCR products from genomic DNA flanking the target sites. The transgenic lines were grown in two greenhouses (I and II) under comparable cultivation environments. Two lines from each greenhouse carrying homozygous deletions of 5 bp in Target1 were predicted to be null mutants and selected for further research (Figure S1b). The genome editing caused a premature stop codon to occur in the first conserved domain of the *DCL2b* protein (Figure S1c). We next predicted there would be four off-target genes for each edited target in the tomato genome using CRISPR-GE^27^. No off-target events were detected at any sites (Figure S2).

### *dcl2b* mutants displayed different phenotypes in two greenhouses

**Fig. 1:**
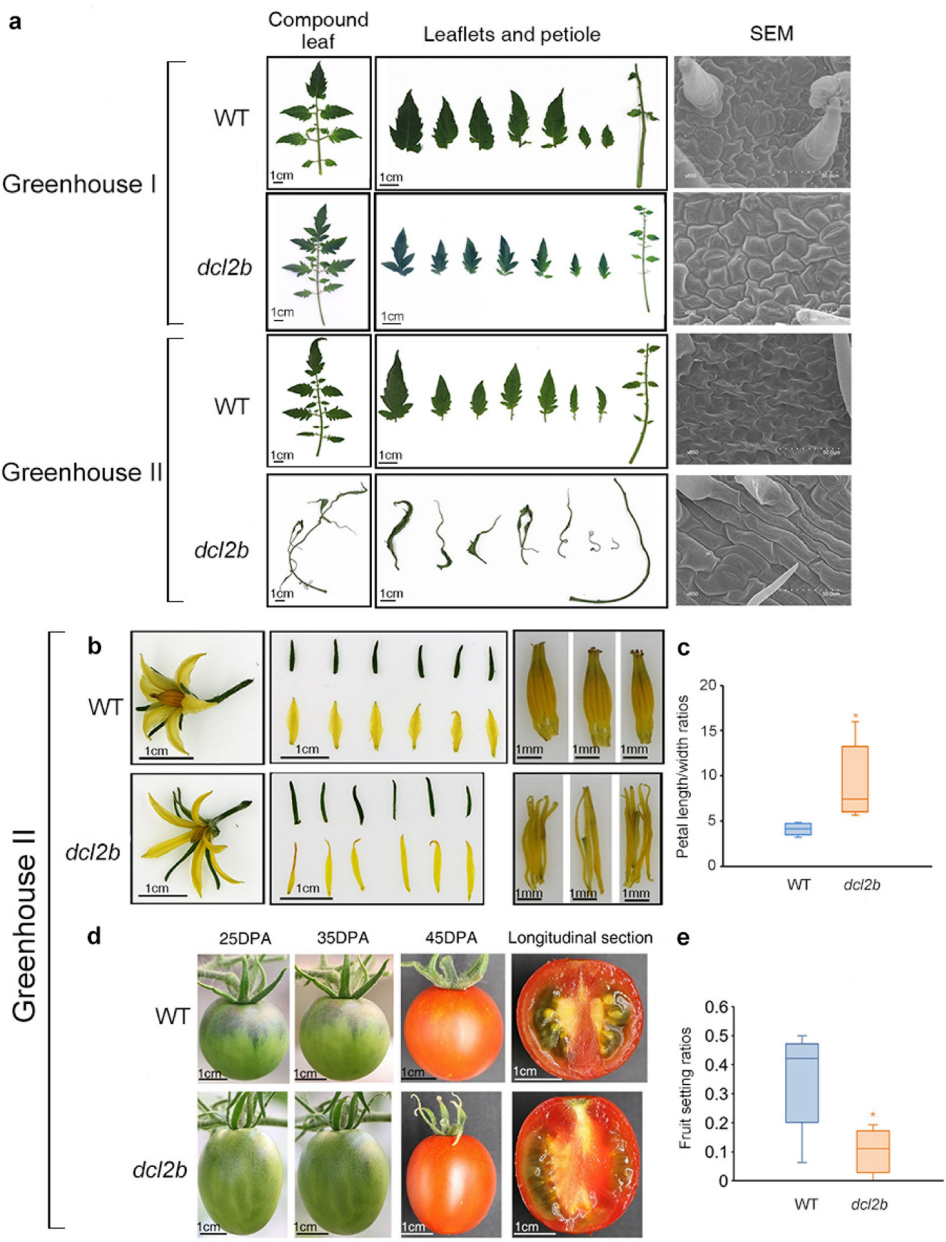
Phenotypes of tomato *dcl2b* mutants. **a** The leaf phenotypes of WT (cv. Ailsa Craig) and *dcl2b* mutants. **b** The flower phenotypes of the WT and *dcl2b* from greenhouse II (from left to right: the whole flower, separated petals and sepals, stamens and pistils). **c** The petal length-to-width ratios of the WT and *dcl2b* from greenhouse II. *N* = 20, asterisks indicate *p* < 0.05 (Student’s *t* test). **d** The fruits of the WT and *dcl2b* from greenhouse II at 25 DPA, 35 DPA and 45 DPA. The longitudinal section of fruits was taken at 45 DPA. **e** Fruit setting ratios of the WT and *dcl2b* from greenhouse II. *N* = 10, asterisks indicated *p* < 0.05 (Student’s *t* test)

In greenhouse I, the tomato WT and *dcl2b* mutants were grown normally without any visible differences (Fig. 1a). However, all the *dcl2b* plants in greenhouse II displayed a strange morphological phenotype compared with the WT. The adult leaves were abnormally long, narrow, and twisted, and the secondary leaflets disappeared. A scanning electron microscope (SEM) analysis of these long leaflets showed that the cells were distinct in size and shape. They became long and columnar rather than irregular polygons (Fig. 1a). In addition to the abnormal leaf development, we observed a similar phenotype in the flowers and fruits of *dcl2b* mutants from greenhouse I. The flowers had more spindly petals and sepals (Fig. 1b, c). We compared the fruits of the WT and *dcl2b* mutant at three stages, at 25 days after pollination (DPA), 35 DPA and 45 DPA. The mutant fruits exhibited an elongated shape whereas the WT fruits were almost round (Fig. 1d). Notably, the fruits of *dcl2b* had few well-developed seeds (Fig. 1d). Furthermore, the fruit setting ratio of the first three branches in the mutant was much lower (Fig. 1e), which might partially result from developmental defects in the stamen and pistil (Fig. 1b).

### Strange narrow pattern phenotype in the *dcl2b* mutant resulting from ToMV infection

**Fig. 2:**
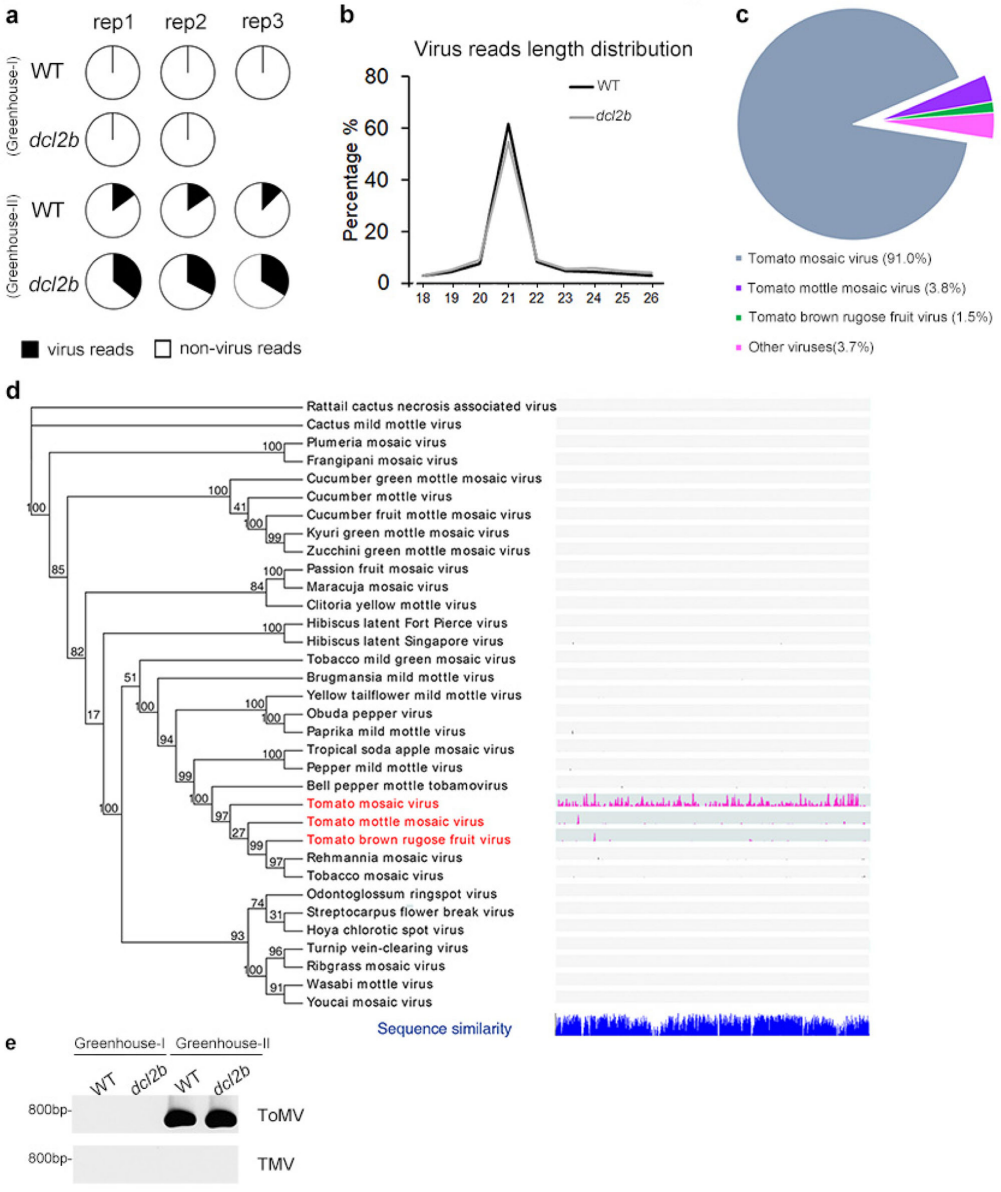
sRNA-seq revealed natural ToMV infections in plants. **a** The percentage of virus-derived reads in the WT and *dcl2b* samples. **b** The length distribution of virus-derived total reads in the WT and *dcl2b* from greenhouse II. **c** The distribution patterns of ToMV, TMMV, TBRFV, and other viruses. **d** Phylogenetic analyses of 34 *Tobamoviruses*. A phylogenetic analysis is shown in the left panel. The three viruses analyzed in the manuscript are marked in red. The genome-wide distributions of virus-derived reads are displayed on the right. **e** An RT-PCR analysis of specific fragments of ToMV and tobacco mosaic virus (TMV)

The strange phenotype was reminiscent of plant symptoms that occur when a viral infection takes place^28^. We wondered whether the morphological phenotype of the *dcl2b* mutants grown in greenhouse II were caused by plant virus(es). Since the deep sequencing of virus-derived small-interfering RNAs (vsiRNAs) has been shown to be an efficient approach for virus discovery in plants and animals^29^, we conducted sRNA-seq with total RNA prepared from the following samples: normal adult leaves from the WT and *dcl2b* from greenhouse I, and normal adult leaves from the WT and “shoestring-like” adult leaves of *dcl2b* from greenhouse II. More than 14 million clean reads ranging from 18 to 26 nt were obtained. Notably, the tomato genome mapping ratios of the WT and *dcl2b* from greenhouse II were obviously lower than those of greenhouse I (Figure S3), suggesting that part of the sequence reads might map to genomes other than that of the tomato. Next, we aligned all the cleaned sRNA-seq reads to 8605 viral genome sequences that were mined from the NCBI (https://www.ncbi.nlm.nih.gov/genome/viruses/). Less than 0.01% of the reads from the WT and *dcl2b* from greenhouse I could be mapped to virus genomes. However, 12.5–35.7% of the reads from greenhouse II matched viral genomes (Fig. 2a).

We analyzed the size distribution of vsiRNAs in the WT and *dcl2b* mutant from greenhouse II and found that more than half of these reads reached 21-nt (Fig. 2b). This result was consistent with previous studies in which the majority of vsiRNAs are 21-nt long in virus-infected plants^20,30,31^. Among the vsiRNAs, over 90% of the reads matched to ToMV, whereas 3.8% and 1.5% belonged to tomato mottle mosaic virus (ToMMV) and tomato brown rugose fruit virus (ToBRFV), respectively, with residue reads aligning to another 8000 viruses (Fig. 2c). The three species are positive-sense single-strand RNA (ssRNA) viruses that belong to the *Tobamovirus* genus^32^.

To examine whether the *dcl2b* mutant was infected with a single ToMV virus or multi-viruses, we checked the distribution of vsiRNAs against 34 total types of tobamovirus RNA genomes from the NCBI GenBank database. The protein sequences of replicases from 34 viruses were aligned and an unrooted neighbor-joining tree was constructed (Fig. 2d). It was obvious that three tomato viruses, ToMV, ToMMV, and ToBRFV, had closer evolutionary relationships. Furthermore, compared with ToMMV and ToBRFV, the vsiRNAs were distributed over the entire ToMV. The few vsiRNA-matched loci in ToMMV and ToBRFV most likely resulted from the high sequence similarity with ToMV. Collectively, these results suggested that the WT and *dcl2b* from greenhouse II were both infected naturally by ToMV. This conclusion was further validated by reverse transcription-PCR (RT-PCR), which showed that a ToMV-specific fragment was detected in the WT and *dcl2b* from greenhouse II, but not in plants from greenhouse I (Fig. 2e). Taken together, these data indicated that the loss of tomato *DCL2b* would increase a plant’s susceptibility to ToMV infection in a natural environment.

To validate the result above, we challenged the tomato WT and *dcl2b* mutants at 2-, 3-, and 4-week stages in a virus-free chamber with ToMV. Again, the *dcl2b* mutants infected by the virus all displayed a strange narrow phenotype while the WT grew normally (Figure S4). This result was fully reproducible in three independent experiments. In addition, an RNA blot showed that the viral RNA accumulation was clearly higher in *dcl2b* than in the WT plants (Figure S4b). Altogether, the developmental defects observed in the *dcl2b* mutant in both the greenhouse and the controlled growth chamber did result from ToMV infection, a further indication that our analysis of datasets collected from naturally and unbiased infection plants are physiologically relevant.

### *DCL2b* was required for the biogenesis of 22-nt sRNA

**Fig. 3:**
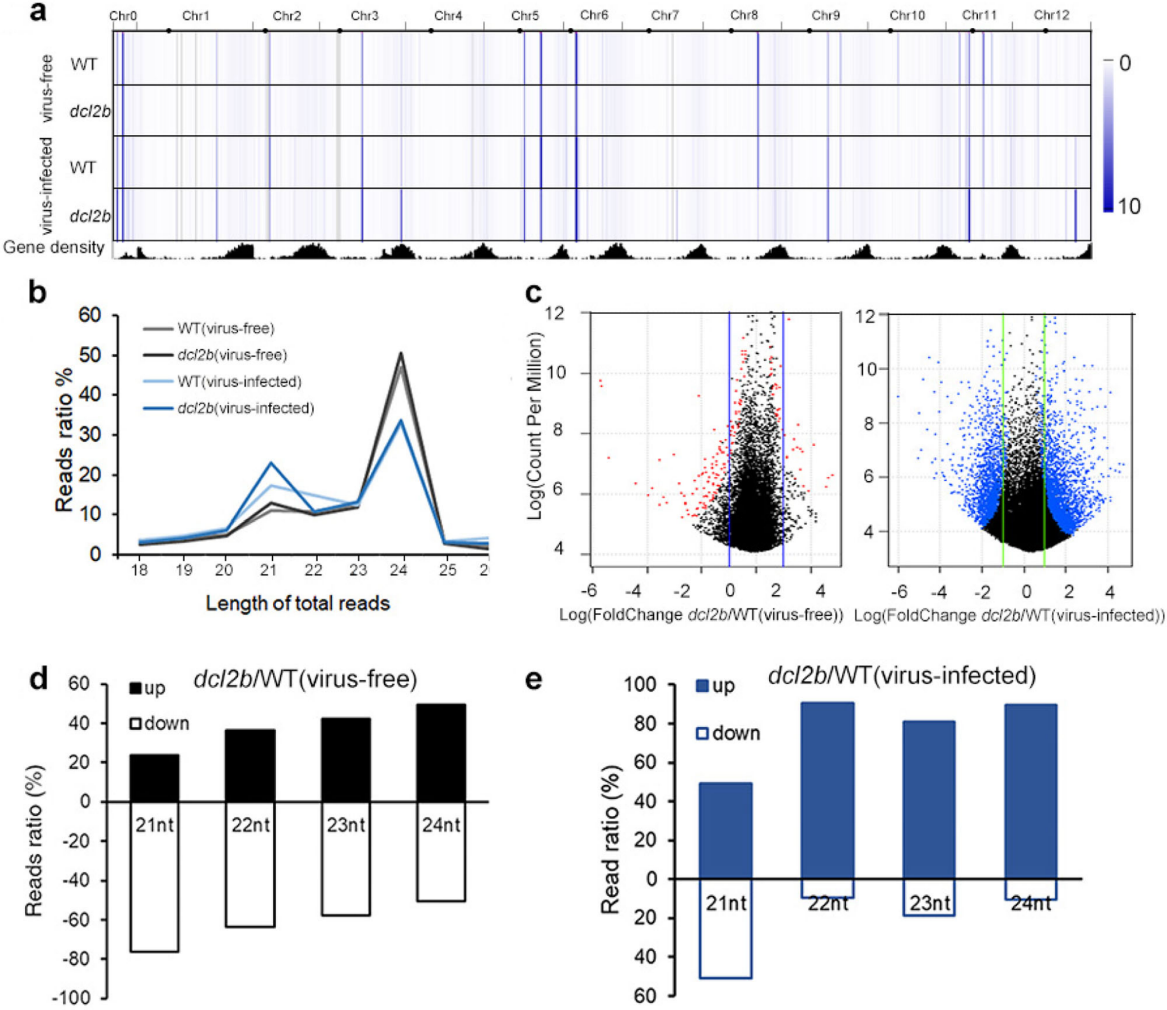
*DCL2b*-affected biosynthesis of tomato 21 and 22-nt sRNAs. **a** A genome-wide heat map of the total sRNA levels. **b** The length distribution of total reads that were mapped to the tomato genome in the WT and *dcl2b*. **c**, Volcano diagrams of differentially expressed sRNAs. Red and blue dots indicate differentially expressed sRNAs in *dcl2b* mutants. **d**, **e** The percentage of tomato 21–24 nt sRNAs that were differentially expressed in *dcl2b* mutants

To investigate the functions of *DCL2b*, we removed the vsiRNAs and focused on endogenous tomato sRNA data for further analysis. Genome-wide profiling by sRNA-seq showed that the tomato sRNAs were not evenly distributed across the chromosomes of the WT and mutants (Fig. 3a). Unlike the tomato long non-coding RNAs^33^, the sRNAs showed higher densities in the pericentromeric heterochromatin regions than in the euchromatin (Fig. 3a). Furthermore, the knockout of *DCL2b* did not influence the overall sRNA distribution (Fig. 3a). Since *DCL2* was previously reported to process dsRNA into 22-nt sRNA^22^, we counted the reads of each sRNA species and found that 22-nt sRNA did not experience a significant decrease in the virus-free *dcl2b* mutant (Fig. 3b). Compared with plants grown in greenhouse I, the populations of 21-nt sRNAs were markedly enhanced in the WT and *dcl2b* from greenhouse II (Fig. 3b). These results were consistent with the previous findings in which parts of these 21-nt sRNAs were novel siRNAs induced by viral infection, and they were designated as virus-activated siRNAs (vasiRNAs)^34^.

We then compared the differentially expressed endogenous sRNAs (DE sRNAs) in two groups. Compared with the WT, 750 sRNAs showed significant change in the *dcl2b* mutant. Among them, 242 (32.3%) were upregulated and 508 (67.7%) sRNAs were downregulated (Fig. 3c). Under viral infection, the number of DE sRNAs rose to 4188, for 3130 (74.7%) increased and 1058 (25.3%) decreased, respectively. These results suggested that the presence of the virus influenced large numbers of sRNAs. To illustrate the role of *DCL2b* in the accumulation of these sRNAs, we separated 21, 22, 23, and 24-nt DE sRNAs and calculated their respective up- and downregulation proportions. Intriguingly, almost 65% of the 21- and 22-nt DE sRNAs were decreased in the *dcl2b* mutant whereas 23- and 24-nt sRNAs showed comparable bilateral changes (Fig. 3d). It has been known that 22-nt sRNAs can trigger the biogenesis of 21-nt sec-siRNAs that are typically produced by *DCL4* in plants^15,16^. Here, we tended to interpret that the production of 22-nt triggers was largely affected in the *dcl2b* mutant, leading to a compromise in the generation of 21-nt sec-siRNAs under virus-free conditions. However, this scenario was not observed under the virus-infection condition since an overwhelming number of DE sRNAs were produced in the absence of *DCL2b* (Fig. 3e).

### *DCL2b* affected miRNA accumulation and sec-siRNA production

**Fig. 4:**
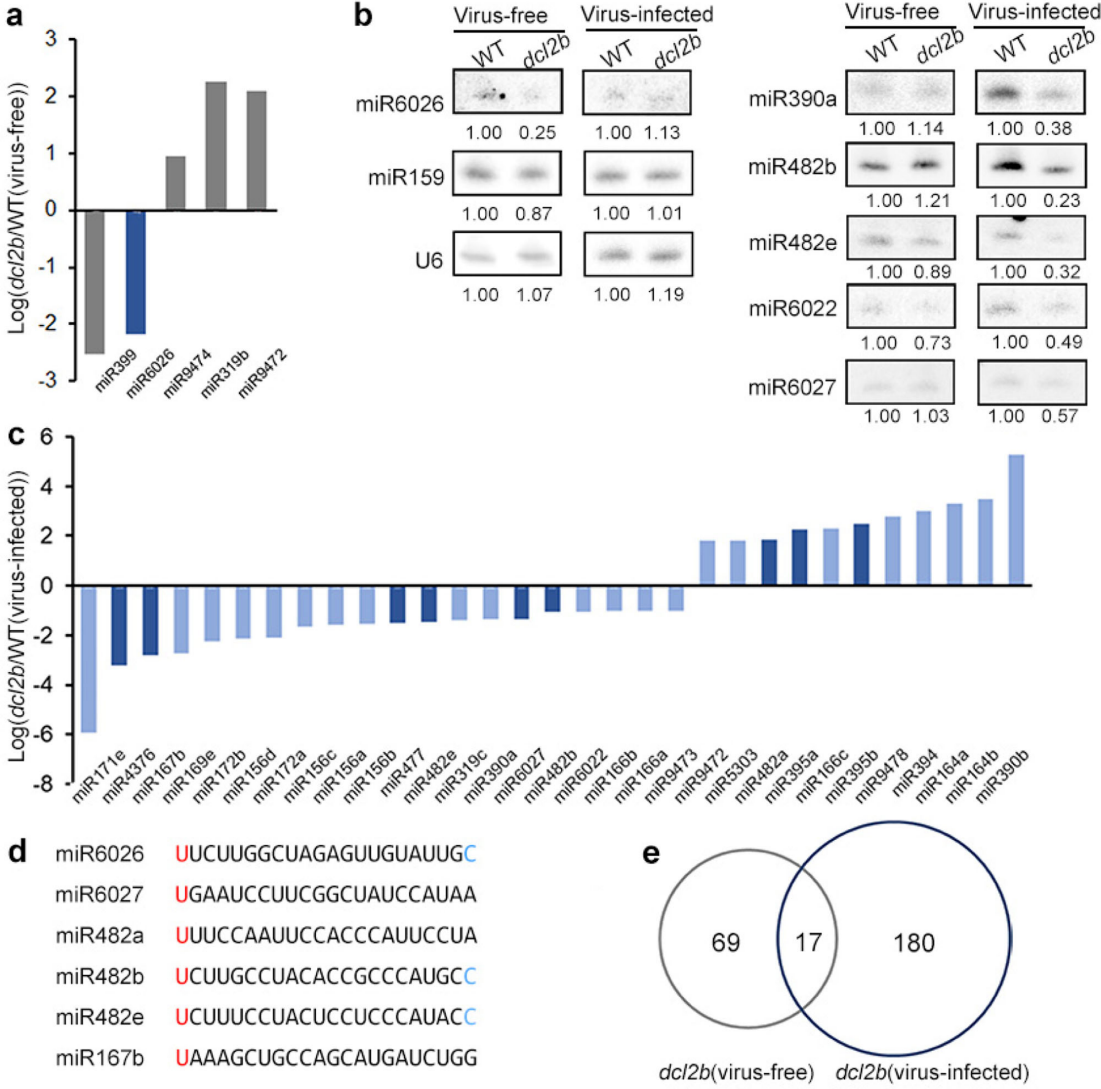
The miRNA levels were influenced in *dcl2b* mutants. **a**, **c** Differentially expressed miRNAs in *dcl2b* mutants. The 22nt miRNAs are colored dark blue. **b** A small RNA blot analysis of miRNAs. **d** The alignment of sec-siRNA triggers. Identical nucleotides and conserved nucleotides among sec-siRNA triggers were marked in red and blue, respectively. **e** Venn diagram for numbers of downregulated precursors of sec-siRNAs in *dcl2b* mutants

Sec-siRNAs can be triggered by 22-nt miRNA. We next examined the impact of tomato *DCL2b* in the regulation of miRNA expression. To this end, we calculated the enrichment levels of 110 mature tomato miRNAs from miRBase. We first examined the miRNA changes under the virus-free condition. Compared with the WT, only five miRNAs exhibited significant changes in the *dcl2b* mutant (Fig. 4a). Notably, the expression levels of 21-nt miR399 and 22-nt miR6026 were reduced. A legitimate target site for miR6026 was predicted in the 5′UTR region of *DCL2a*, *2b*, and *2d*, raising the possibility that miR6026 might act as a trigger for sec-siRNA biogenesis from the three *DCL2* genes^7^. In the virus-infected *dcl2b* mutant, the number of DE miRNAs was elevated to 31, and the expression of DE miRNAs was easily validated by a small RNA blot analysis (Fig. 4b). Importantly, 9 of the 31 DE miRNAs reached 22-nt (Fig. 4c). Interestingly, we aligned six 22-nt DE miRNA sequences and found that they all had a U in the 5′ position and three miRNAs had a C at the 3′ terminal nucleotide (Fig. 4d), indicating that these miRNAs were likely associated with the initiation of sec-siRNA production^16^.

To investigate whether *DCL2b* contributed to the sec-siRNA production through 22-nt miRNAs, we aligned all the sRNAs measuring 18–26-nt to tomato transcripts (genome annotation ITAG3.2), revealing perfect matches using Bowtie and an in-house Perl program. We named the transcripts that served as the precursors of sec-siRNAs as templates, and then we extracted the templates that contained less than half the reads of sec-siRNAs generated in *dcl2b* mutants compared with their own WT controls. Here, we predicted 96 and 197 potential templates in virus-free and virus-infected *dcl2b*, respectively, with 17 overlaps (Fig. 4e and Table S1). In the previous studies, *DCL4*, together with *AGO1*, *AGO4*, *AGO7*, *SUPPRESSOR OF GENE SILENCING3* (*SGS3*), *RDR6*, and *DOUBLE-STRANDED RNA BINDING FACTOR 4* (*DRB4*), were found to participate in different steps of sec-siRNA production^15,35^. To investigate if these above genes had any impact on the sec-siRNA production from the above filtered templates, we measured their abundance according to RNA-seq data that were obtained from the same sets of samples for the sRNA-seq above. We obtained ~20 million clean reads from RNA-seq, over 80% of which could align to the tomato genome (with two mismatches) (Figure S3). We found that none of these sec-siRNA-related genes displayed obvious changes in transcription when *DCL2b* was knocked out (Figure S5a). Thus, the differential accumulation of sec-siRNAs is unlikely caused through the indirect impact of these sec-siRNA-related genes.

**Fig. 5:**
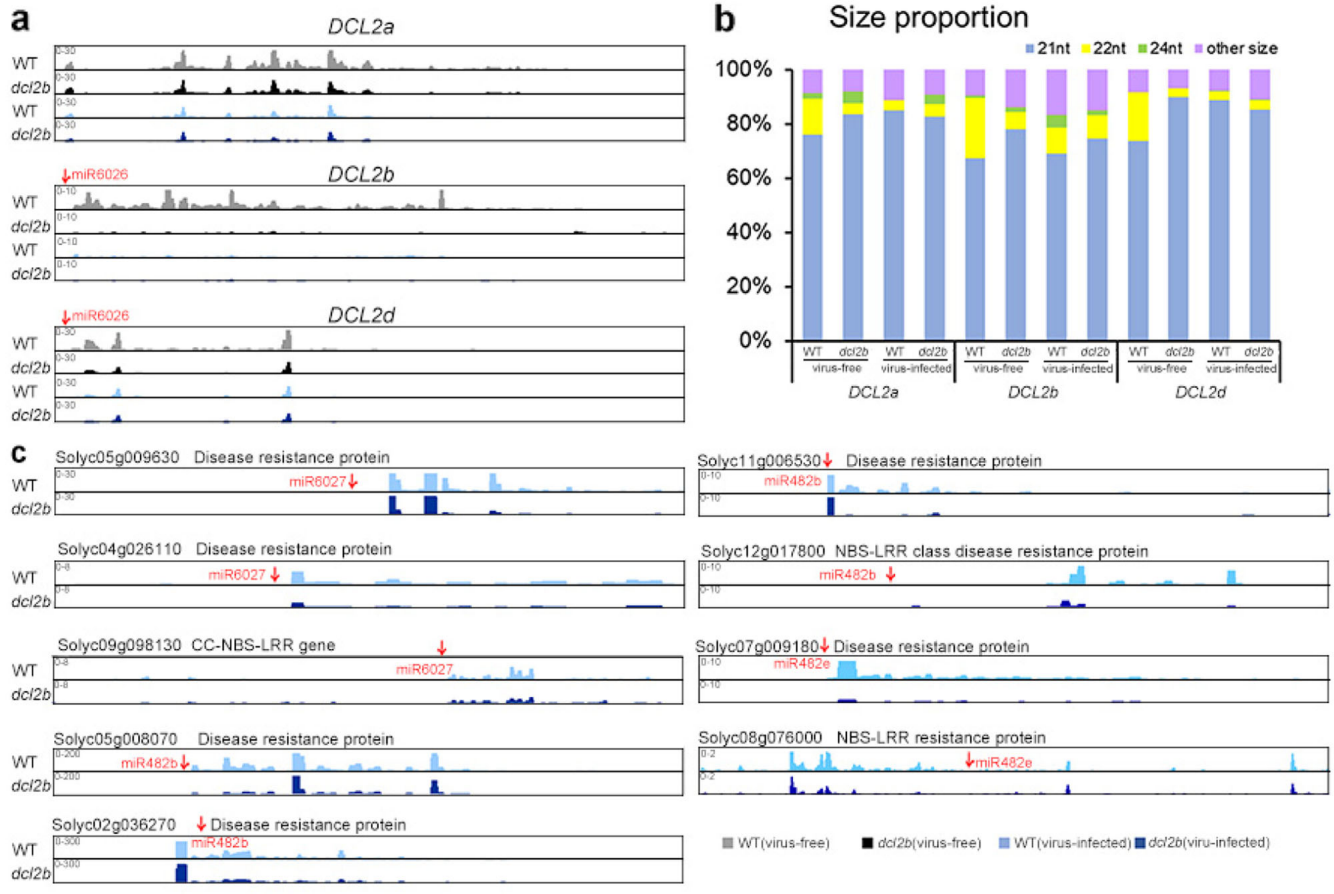
Sec-siRNAs triggered by 22-nt miRNAs. **a** The sec-siRNA levels of *DCL2a*, *2b* and *2d* are shown with screenshots from Integrative Genomics Viewer (IGV). For the display, the three biological replicates of each sample were merged. Red arrows show the positions of sites complementary to miR6026. The horizontal axes represent the sRNA reads per million (RPM). **b** The size distribution of sec-siRNAs that are produced from *DCL2a*, *2b* and *2d*. **c** The sec-siRNA levels of nine genes triggered by miR6027, miR482b, and miR482e are shown with screenshots from IGV. For display clarity, three biological replicates of each sample were merged. The arrows indicate the sites complementary to miRNAs. The horizontal axes represent the sRNA RPM

In the virus-free WT and *dcl2b* mutant, we hypothesized that the 22-nt miR6026 might lead to the formation of sec-siRNA from targeted mRNAs such as *DCL2a*, *2b*, and *2d* (Figure S5b). The expression level of miR6026 in the *dcl2b* mutant decreased significantly (Fig. 4b and Figure S5c). The sec-siRNA abundance from three *DCL2* transcripts was reduced two- to seven-fold (Fig. 5a and Figure S5d). Intriguingly, over 70% of the total sec-siRNAs measured 21-nt (Fig. 5b), suggesting that *DCL4* might play a major role in 21-nt sec-siRNAs biosynthesis from the *DCL2* transcripts. It is noteworthy that the formation of 22-nt sec-siRNA was suppressed in the *dcl2b* mutant, suggesting that *DCL2b* is also involved in the biogenesis of 22-nt sec-siRNAs (Fig. 5b). Next, we used Integrative Genomics Viewer (IGV) to illustrate the above results. Normalized sRNAs were mapped to three *DCL2* loci, and the IGV screenshots clearly showed that sec-siRNAs were produced in a phased pattern of each locus (Fig. 5a). We could also note that the sec-siRNAs were less abundant in the *dcl2b* mutant (Fig. 5a). The miR6026 expression did not change significantly in the virus-infected *dcl2b* mutant (Figure S5c). As a consequence, the abundance variations in *DCL2*-derived sec-siRNAs were not as obvious as those in the virus-free samples (Fig. 5a and Figure S5d).

In contrast to virus-free plants, more 22-nt miRNAs experienced significant decreases in the virus-infected *dcl2b* mutant (Fig. 4a, c). We selected three downregulated ones for analysis to find if they acted as triggers of sec-siRNAs. Notably, three, four, and two genes had target loci for miR6027, miR482b, and miR482e, respectively, all of which are disease-related genes. The sec-siRNAs generated from these templates were remarkably reduced in the tomato *dcl2b* mutant (Figure S5e). The IGV screenshots also illustrated the phased patterns of sec-siRNAs (Fig. 5c), indicating that the three miRNAs caused target slicing and phased siRNA production. Furthermore, when we checked the annotations of the sec-siRNAs precursor from *dcl2b*, we found 66 out of 197 precursors belonged to the “disease resistant” category (Table S1), revealing *DCL2b*’s critical function in the sec-siRNA biogenesis after ToMV infection. From the above results, we could conclude that *DCL2b* plays an important role in sec-siRNA production. When tomato plants are infected by ToMV, *DCL2b* plants have an even broader impact on disease-resistant gene-derived sec-siRNAs, at least partially through their 22-nt sRNA triggers.

### Function analysis of *DCL2b*-activated and -repressed genes

**Fig. 6:**
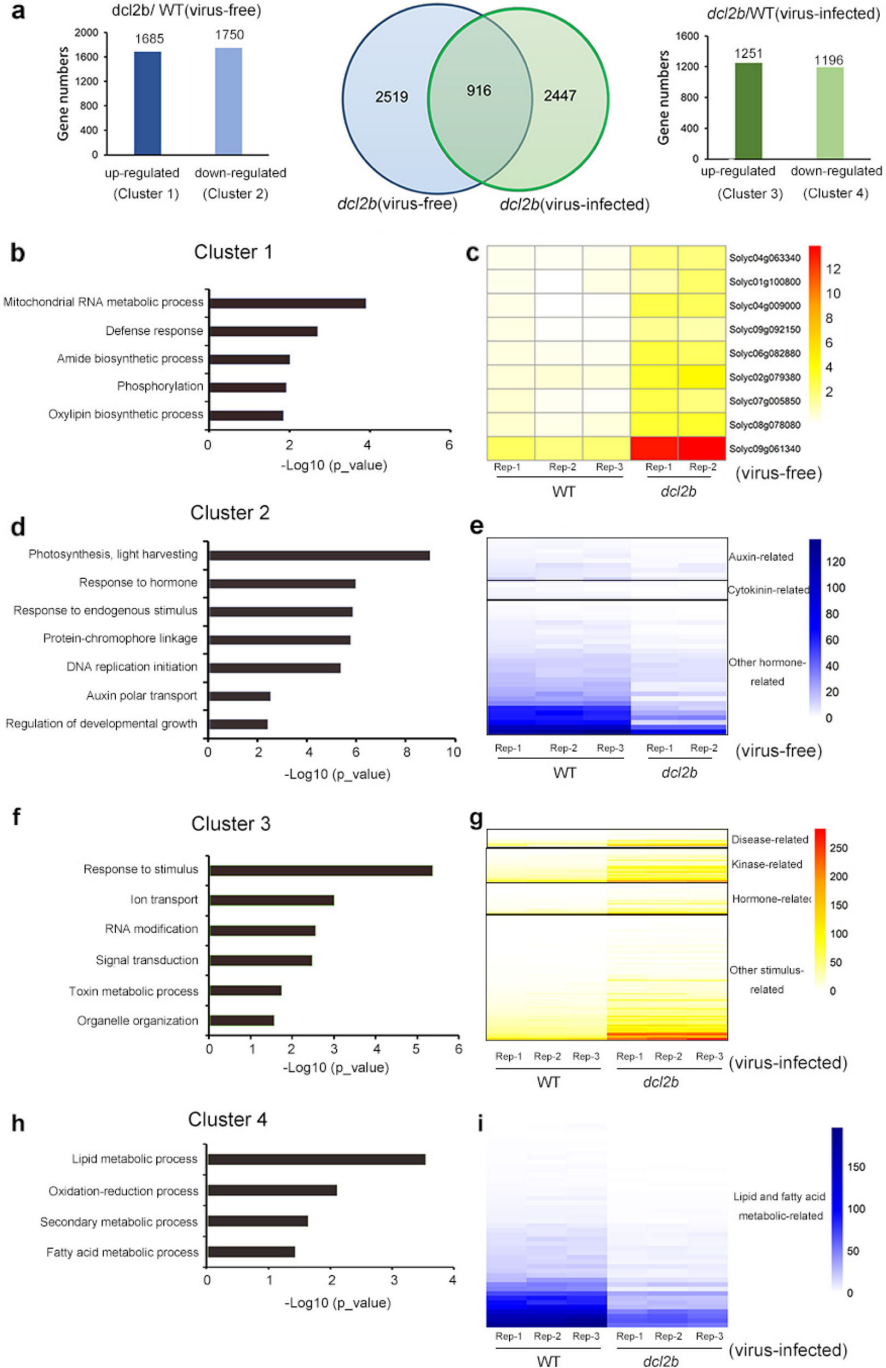
Gene Ontology enrichment analysis for the differentially expressed genes. **a** Venn diagrams for numbers of DEGs in the *dcl2b* mutant. The left panel shows up- (Cluster 1) and downregulated (Cluster 2) genes in virus-free *dcl2b*. The right panel shows up- (Cluster 3) and downregulated (Cluster 4) genes in virus-infected *dcl2b* after excluding the overlapped 916 transcripts. **b**, **d**, **f**, **h** GO enrichment analysis of Cluster 1-Cluster 4 genes. **c**, **e**, **g**, **i** Heat map of genes enriched during the mitochondrial RNA metabolic process, hormone signaling, response to stimulus, and lipid and fatty acid metabolism pathways, respectively

To further investigate the function of tomato *DCL2b*, we examined the global expression profiles of the WT and *dcl2b* plants through RNA-seq. Compared with the WT, we identified 3435 and 3363 genes that were differentially expressed (DEGs) (|log2(FoldChange)| > 1, *p*-value < 0.05) in virus-free and virus-infected *dcl2b* mutants, respectively, of which 916 transcripts were overlapped (Fig. 6a middle). In the virus-free *dcl2b* mutant, 1685 transcripts were elevated, whereas 1750 were reduced (Fig. 6a left), and these DEGs might merely represent the *DCL2b* function under the normal growth condition, whereas DEGs in the virus-infected *dcl2b* would partially result from the virus infection. If we excluded the 916 overlapped transcripts from the total DEGs, the remaining 2447 DEGs were more likely related to defense against viruses. Among the 2447 DEGs, there were 1251 upregulated genes and 1196 downregulated transcripts in the *dcl2b* mutant (Fig. 6a right).

To understand the biological processes associated with *DCL2b*-related or virus-affected genes, DEGs were divided into four clusters (upregulated in virus-free *dcl2b* (1), downregulated in virus-free *dcl2b* (2), upregulated in virus-infected *dcl2b* (3), and downregulated in virus-infected *dcl2b* (4)), and then they were subjected to Gene Ontology (GO) analysis separately (Table S2). For Cluster 1, the genes involved in the mitochondrial RNA metabolic process and the defense response were highly enriched (Fig. 6b). We tested the expression of nine genes in the mitochondrial RNA metabolic process pathway and found that all of them belonged to the pentatricopeptide repeat (PPR) protein family (Fig. 6c). These PPR genes have a range of essential functions in post-transcriptional processes within the mitochondria and chloroplasts^36^. Our GO analysis showed that Cluster 2 genes were highly enriched in a hormone metabolism pathway (Fig.  6d). We examined 41 genes that are related to auxin, cytokinin, and other hormones such as gibberellic acid (GA) and found that they were reduced significantly (Fig.  6e).

Next, we performed a GO enrichment analysis of virus-induced genes in Cluster 3. As shown in Fig. 6f, genes associated with responses to stimuli were highly enriched. Of all 145 transcripts, 12, 27, and 22 genes belonged to disease-, kinase-, and, hormone-related categories, respectively (Fig. 6g), suggesting that these genes could play an important role in responding to ToMV infection. For Cluster 4, plenty of DEGs were related to the lipid and fatty acid metabolic pathway (Fig. 6h). When viruses invade a cell to complete the replicative cycle, they will express their own proteins and co-opt host cell factors for multiplication, including lipids^37^. The initial steps include the attachment to a specific receptor, in some cases a specific lipid. The replication of the viral genome then takes place, either in association with cellular membranes or other lipid structures. Next, new virus genomes are enclosed inside synthesized viral particles, in which many lipids also play an important role^38^. Since host lipids are essential for multiple steps of the viral replication cycle, plants can use different strategies to interfere with viral infection. One of the strategies is to reduce the lipid metabolism and biogenesis, which might explain why the lipid-related genes were downregulated in *dcl2b* (Fig. 6i).

## Discussion

Here, we systematically studied the function of tomato *DCL2b*. Generally, *DCL2b* protein might have a role comparable to that of *Arabidopsis* *DCL2* in producing 22-nt sec-siRNAs. However, this very protein also has some unique functions in the biogenesis of 22-nt miRNAs, which in turn cause the production of sec-siRNAs. This function has typically been assigned to *DCL1* in *Arabidopsis*. In addition, the tomato *DCL2b* appears to play a more important role than *Arabidopsis DCL2* in the defense against viruses.

### Tomato *DCL2b* was responsible for sec-siRNA biogenesis

In *Arabidopsis*, 22-nt sRNAs are the key determinant of sec-siRNA triggers^16^. *DCL2* usually generates 22-nt siRNAs from perfectly duplexed precursors^21^. For 22nt-miRNA, its precursor contains a bulge, so *DCL1* generates a 22-nt miRNA and a 21-nt miRNA*^16,39^. In our research, we found that tomato *DCL2b* was required for the biogenesis of 22-nt siRNAs, which served as triggers to generate sec-siRNAs (Fig. 3d and Table S1). Notably, the level of 22-nt miR6026 decreased significantly in the *dcl2b* mutant (Fig. 4a, b). In a previous report, miR6026 was not processed by tomato *DCL1*^7^. Moreover, its precursor does not contain asymmetric bulges (miRBase: http://www.mirbase.org/), raising the possibility that miR6026 is *DCL2b*-dependent. Additionally, miR6026 was predicted to target tomato *DCL2a*, *2b*, and *2*d^7^. In our study, we also discovered that miR6026 could trigger sec-siRNAs from these three *DCL2* transcripts (Fig. 5a), suggesting that there may be a feedback regulation mechanism between *DCL2b* and miR6026.

When tomato plants were infected by ToMV, a large number of 21-nt sRNAs were markedly enhanced (Fig. 3b, c). The expression levels of *DCL2b* accumulated as well, both in the WT and *dcl2b* mutant (Figure S4c and S6). The above feedback loop was destroyed and miR6026 was not decreased in the virus-infected *dcl2b* mutant (Fig. 4b). Other 22-nt miRNAs showed downregulation (Fig. 4c). *DCL2b* might not participate in their processing directly but could influence their expressions when the tomato was infected by a virus. These 22-nt miRNAs acted as sec-siRNA triggers, and all of them targeted disease-related genes (Fig. 5c). In the tomato, *Tobacco mosaic virus resistance-1* (*Tm-1*) encodes a protein that can bind to ToMV replication proteins and inhibit its replication^40,41^. Due to its low expression (RPM < 1), we could not determine if *Tm-1* serves as a sec-siRNA precursor. However, when we calculated the precursors that generated less sec-siRNAs in *dcl2b*, we found 197 transcripts, and one-third of them belonged to the “disease-resistant” category (Fig. 4e and Table S1), suggesting that the tomato *DCL2b* appears to regulate disease-related genes by processing sec-siRNAs while combating ToMV.

### Tomato *DCL2b* played a key role in defending against ToMV

In *Arabidopsis*, *DCL4* and *DCL2* act hierarchically in antiviral resistance^24^. *DCL4* is considered the leader and is primarily responsible for the processing of 21-nt vsiRNAs from RNA viruses^20^. Only when *DCL4* is absent or its activity is suppressed by viruses does *DCL2* produce 22-nt vsiRNAs, serving as a backup to functionally compensate for *DCL4*^19,42^. Turnip crinkle virus (TCV) is an example because this virus encodes a suppressor P38 that specifically inhibits *DCL4* activity. In this scenario, *DCL2* contributes to a majority of vsiRNAs^19^. For other viruses such as Potato virus X (PVX), *DCL4* alone is sufficient to inhibit PVX accumulation. The infected *Arabidopsis dcl2* mutant does not show viral symptoms^43^. During Turnip mosaic virus (TuMV) infection in *Arabidopsis*, *DCL4*-dependent siRNAs are necessary to prevent initial infections whereas *DCL2* is neither necessary nor sufficient to limit infections^20^. In our study, when the tomato *DCL2b* itself was knocked out, the plants showed severe developmental defects both in natural and manual ToMV infection environments (Fig. 1a, S4a and S4d). This scenario has not been observed with *Arabidopsis DCL2*, implying that the tomato *DCL2b* has more important roles in virus defense compared to its *Arabidopsis* counterpart. When the tomato was infected with ToMV, the *DCL4* expression remained unchanged (Figure S4c and S6). The 21-nt vsiRNAs and tomato endogenous sRNA also did not decrease (Fig. 2b), suggesting that *DCL4* was still functional after antiviral silencing. The ToMV virulence in tomatoes is very strong, and plants need both *DCL2b* and *DCL4* to combat the virus. Together, these results indicate that plants might be regulated by unique mechanisms during different species–virus interaction.

## Materials and methods

### Plant materials and growth conditions

The tomato wild type (cv. Ailsa Craig) and mutants (with AC background) were planted in commercial tomato cultivation soil. All the plants were grown in two greenhouses under the same conditions, 20–25 °C under a 16 h light/8 h dark cycle.

### CRISPR/Cas9 gene knockout and mutation analysis

Four sgRNAs targeting four exons of *DCL2b* were designed using the online tool CRISPR-GE (http://skl.scau.edu.cn/home/). These 20 bp oligos were cloned into AtU3d, AtU3b, AtU6-1, and AuU6-29 vectors, respectively. The above sgRNA expression cassettes were assembled into pYLCRISPR/Cas9-Ubi-H binary plasmid by Golden Gate ligation^44^. Tomato tissue culture was performed according to the established protocol using the *Agrobacterium* infection method^45^. For the mutation analysis, genomic DNA was extracted from young tomato leaves using a Plant Genomic DNA Kit (Tiangen, China). The DNA was used as a template to amplify the *DCL2b* fragment using PCR. The fragments were then sent out for sequencing. The primers used in the vector construction and mutation analyses are listed in Table S3.

### Virus inoculation procedures

Viral inoculations were performed as described^46^. The tomato plants were infected at 2-, 3-, and 4-week stages and the inoculated plants were placed in the incubator at 22 °C.

### High-throughput sequencing of RNAs and sRNAs

The total RNA samples were prepared from WT and *DCL2b* mutant adult leaves using TRIzol reagent (Invitrogen, USA). Paired-end mRNA libraries were generated using NEBNext^®^ Ultra^TM^ RNA Library Prep Kit for Illumina^®^ (NEB, USA) according to the manufacturer’s recommendations and were sequenced on an Illumina HiSeq 4000 platform; 150 bp reads were generated. The quality of clean reads was checked using the FastQC program (v0.11.3). Then, Fastq data were aligned to the tomato genome (SGN release version SL3.0) using TopHat (v2.1.0). These mapped reads were counted by HTSeq (v0.6.1) and differential expression analysis was performed using the DESeq2 package. sRNA libraries were prepared using NEBNext^®^ Multiplex Small RNA Library Prep Set for Illumina^®^ (NEB, USA.) and sequenced on an Illumina HiSeq2500 platform, and 50 bp single-end reads were generated. The quality of clean reads was also checked by FastQC. Subsequently, FASTA data were aligned to the virus genomes from NCBI (ftp://ftp.ncbi.nlm.nih.gov/refseq/release/viral/) using Bowtie (v1.1.2). The unmapped reads were then aligned with the tomato genome. A differential expression analysis was processed with an in-house python script.

### RNA extraction, real-time PCR analysis, and northern blot

The total RNA was extracted with TRIzol reagent from the tomato leaves (Invitrogen, USA). For reverse transcription, 1 μg of RNA and oligo dT primers were used to synthesize cDNA using a TranScript One-Step gDNA Removal and cDNA Synthesis SuperMix kit (Trans, China). Real-time PCR was then performed on a CFX96 Real-Time PCR Detection System (Bio-Rad, USA) with SYBR Green PCR Master Mix (Trans, China). *Actin* was used as an internal control. Each experiment included three independent biological repeats and three technical replicates.

For the ToMV northern blot, 10 μg of total RNA was used in each lane. Hybridizations were performed with biotin-labeled probes complementary to the ToMV sequence. Loading RNA served as the control. For the small RNA northern blot, 10 μg of total RNA was used in each lane. Hybridizations were performed with 32^P^-radiolabeled probes complementary to miR159, miR390a, and miR6022. U6 served as the loading control^6^. The primers and probes used in the real-time PCR analysis and northern blot are listed in Table S3.

### Imaging SEM

For SEM analysis, leaf samples were first fixed in 2.5% glutaraldehyde buffer with 0.1 mol/L sodium phosphate (pH 7.2) for 2 h. After the samples were dehydrated with serial ethanol washes, they were dried with a critical point dryer and then coated with gold particles. The samples were examined with an SEM (HITACHI S-3400N, Japan)^47^.

### Gene Ontology analysis

Gene Ontology enrichment analysis was performed using Gene Ontology Consortium online tools^48^. The genes were calculated by PANTHER overrepresentation method. Binomial tests with Bonferroni correction were used to calculate the *p*-values. Terms with *p*-values < 0.05 were considered to be enriched.

### Phylogenetic analysis

Protein sequences from 34 *Tobamovirus* were aligned using the ClustalX 2.1 with default parameters^49^ and a phylogenetic tree was constructed through MEGA6.06 by neighbor-joining method with 1000 bootstrap replicates and visualized with the online tool Evolview^50^.

### Statistical analysis

SPSS statistics (v19.0) was used for the statistical analysis. The statistical significance was computed using Student’s *t* test. Significant differences (*p* < 0.05) were indicated by asterisks. All data were presented as the means ± standard errors (SEs).

### Data availability

Raw data from RNA-seq and sRNA-seq have been submitted to the Sequence Read Archive (SRA) at NCBI (http://www.ncbi.nlm.nih.gov/sra/) under the accession number SRP136048.

## Electronic supplementary material

Supplementary figures

Dataset 1

Dataset 2

Dataset 3
