# Supplementary material for: Tomato DCL2b is required for the biosynthesis of 22-nt small RNAs, the resulting secondary siRNAs, and the host defense against ToMV
Source: Hortic Res. 2018 Sep 1;5:62. doi: 10.1038/s41438-018-0073-7 (PMC6119189; doi:10.1038/s41438-018-0073-7)
Supplement: Supplementary file 1 — Supplementary figures [file 41438_2018_73_MOESM1_ESM.docx]

Figure S1


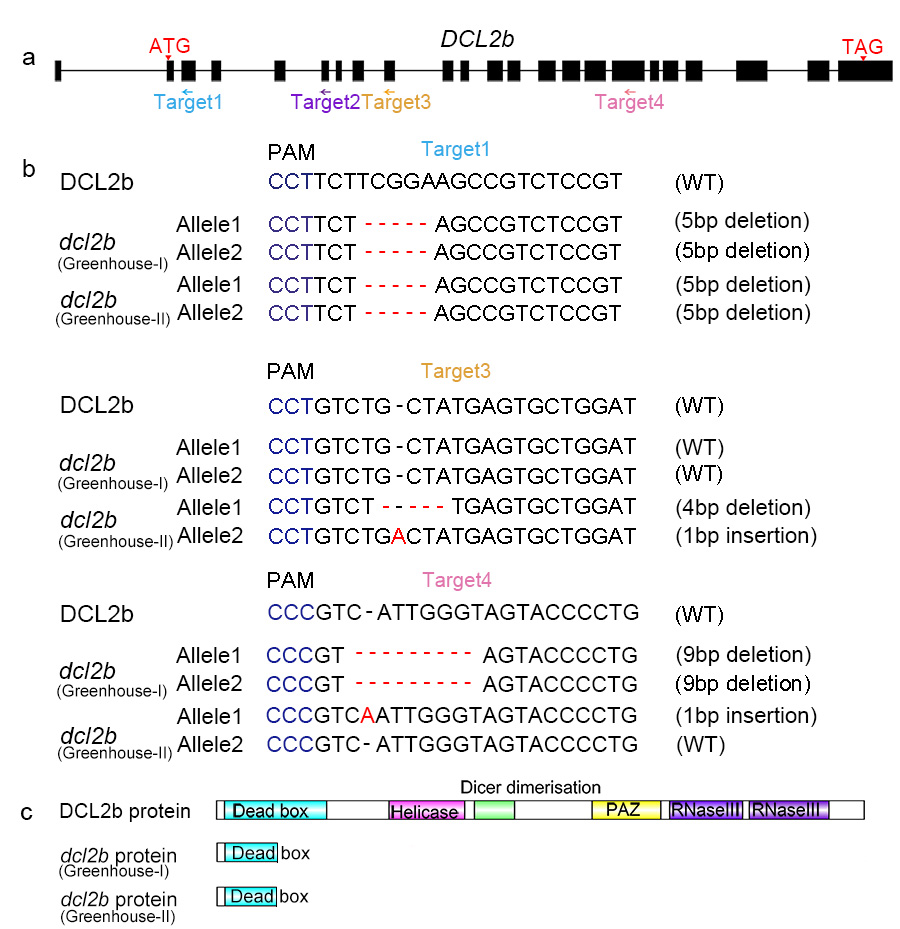


**Figure S1. Genome editing of *DCL2b* in tomato plants.**

**a**, Schematic illustration of four targets in *DCL2b* genomic sequence. **b**, Genotyping of mutations in *dcl2b* mutants. PAM, protospacer adjacent motif. Red dashed lines indicated deletion. Red letters indicated insertion. **c,** Predicted peptide domain analysis of DCL2b proteins.

Figure S2


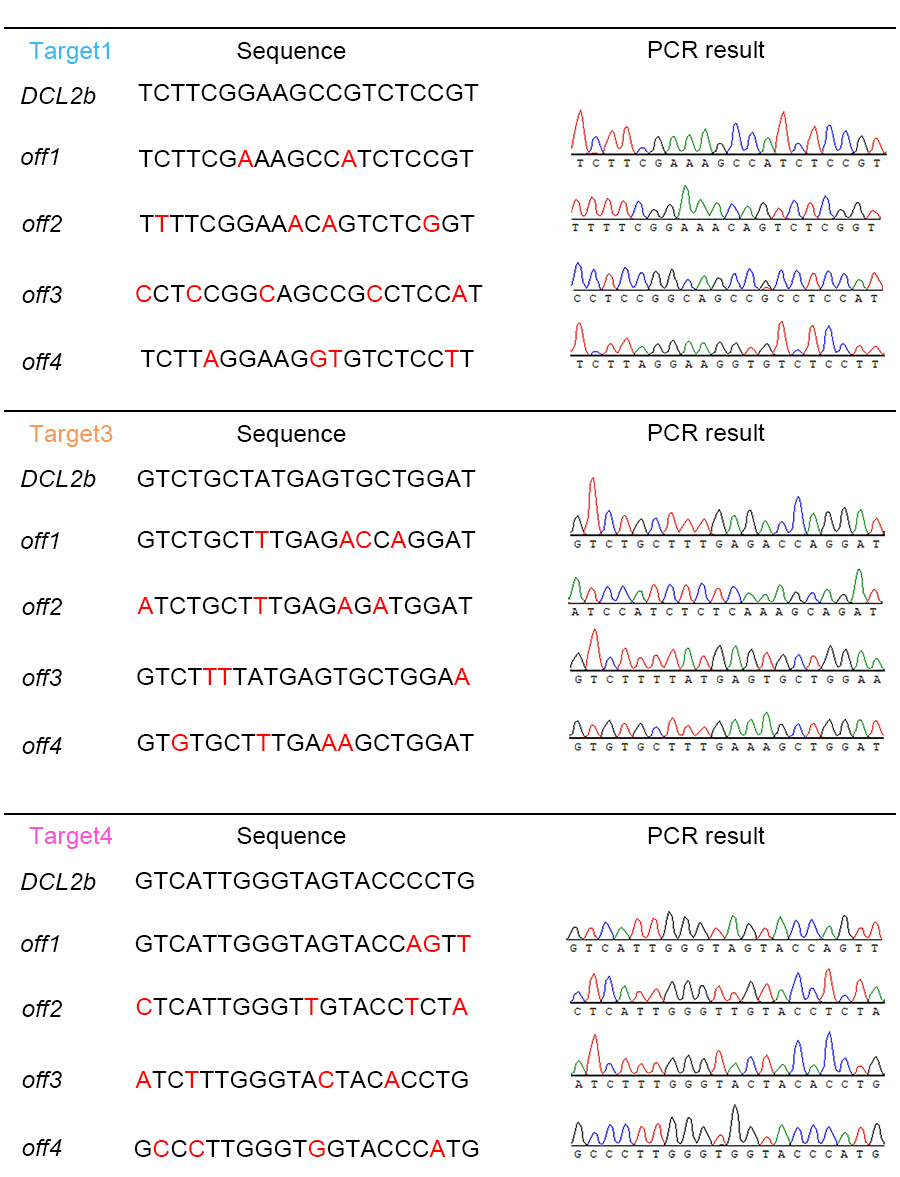


**Figure S2.** Off-target detection in *dcl2b* mutants.

Mismatching bases were marked in red.

Figure S3


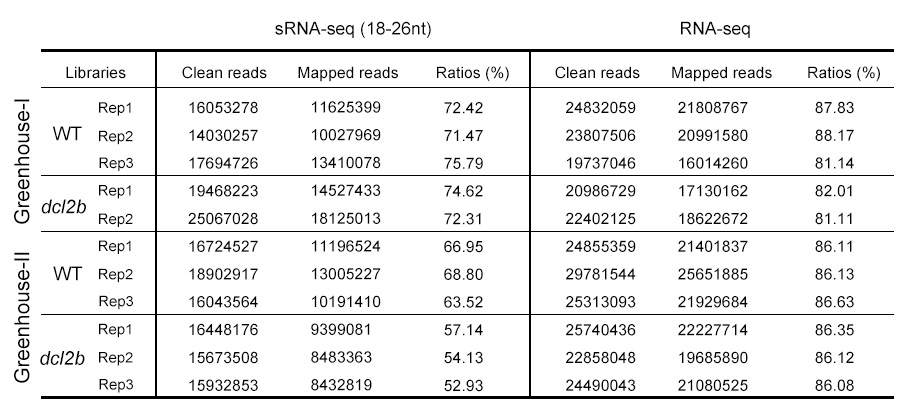


**Figure S3**. Summary of clean reads mapping ratios of sRNA-seq and RNA-seq data sets.

Figure S4


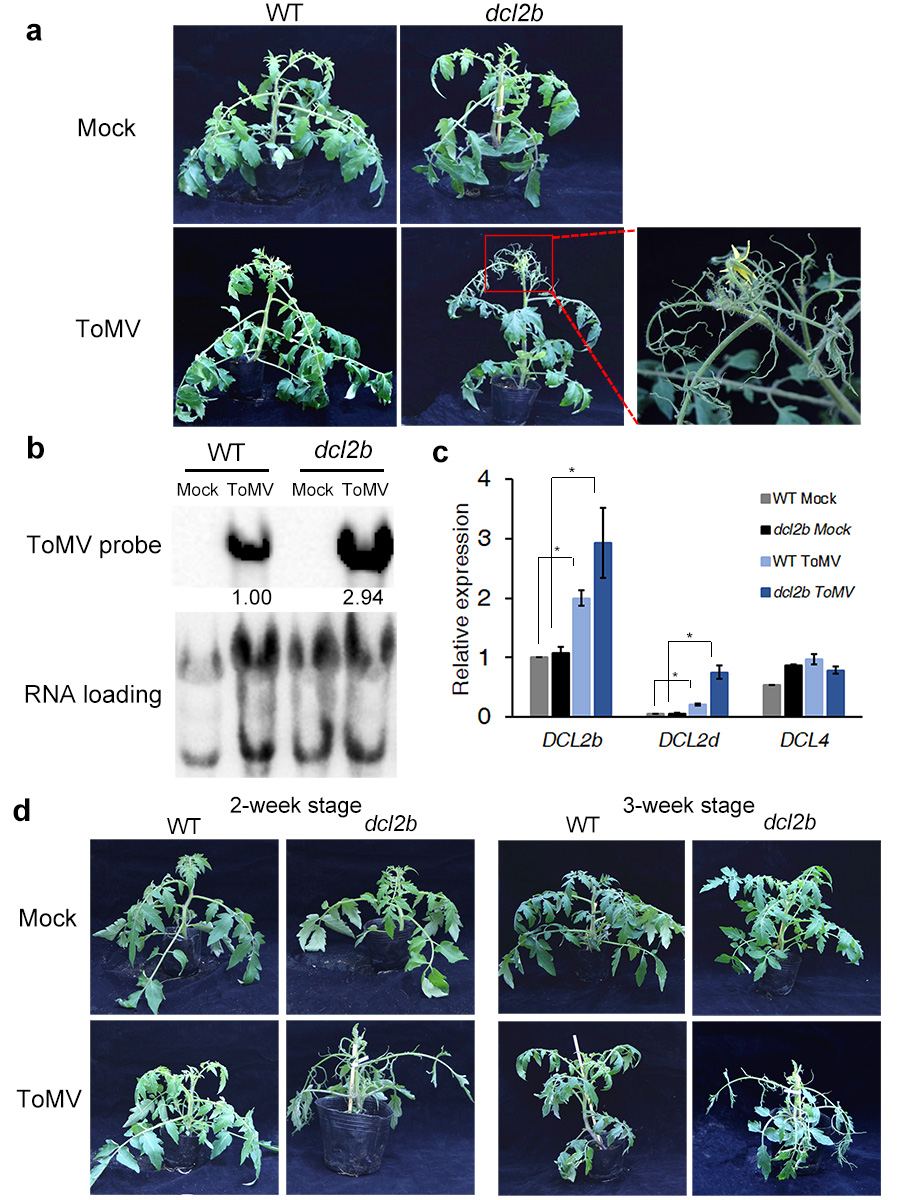


**Figure S4.** The *dcl2b* mutant infected manually with ToMV showed the identical developmental defects observed in the naturally-infected *dcl2b* mutant.

**a**, WT and *dcl2b* mutant inoculated with ToMV at 4-week stage. The red boxed area represented enlargement of weird leaves. **b**, Northern blot analysis of ToMV titers. **c**, Relative expression of *DCL2b*, *DCL2d* and *DCL4* in WT and *dcl2b* when infected by ToMV. Asterisks indicated p<0.05 (Student’s *t* test). **d**, WT and *dcl2b* mutants inoculated with ToMV at 2- and 3-week stages.

Figure S5


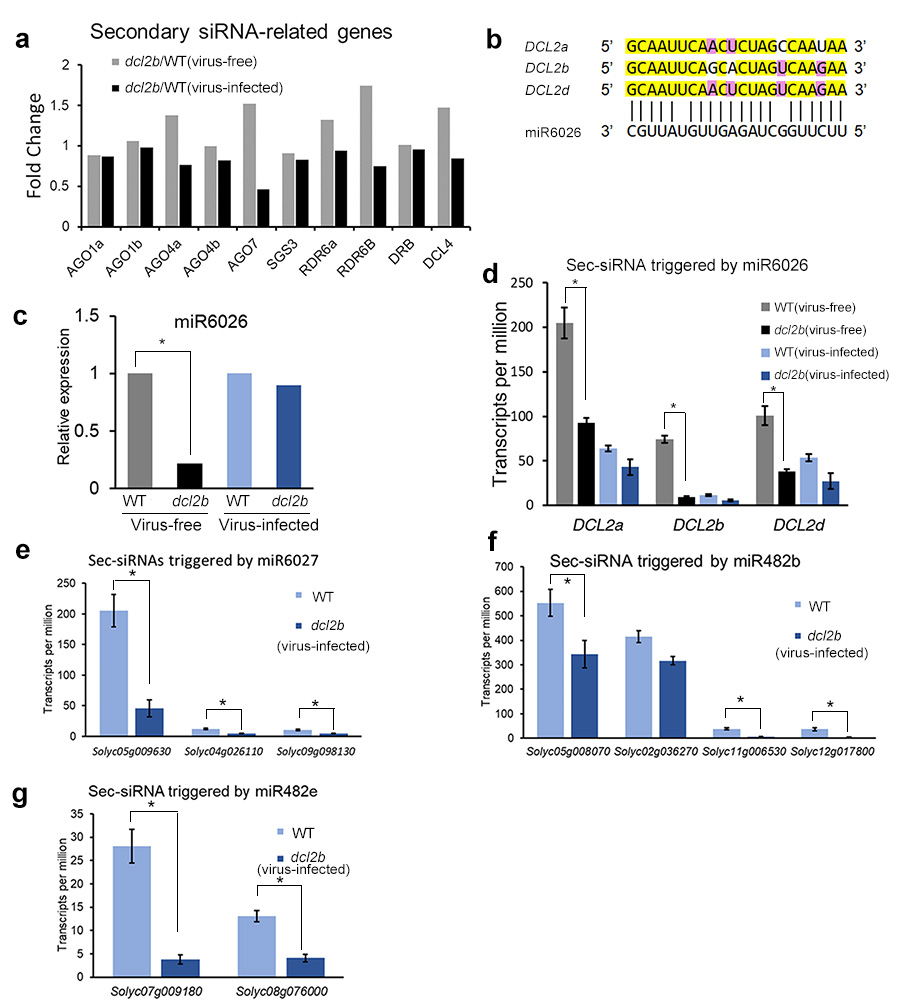


**Figure S5.** Sec-siRNAs triggered by 22-nt miRNAs.

**a**, Fold change of sec-siRNA production-related genes. **b**, Sequence alignment between *DCL2* genes predicted to serve as miR6026 targets. **c**, The expression of miR6026 in WT and *dcl2b* mutants. **d**, The abundance of sec-siRNAs that produced from *DCL2a*, *2b*, and *2d*. **e**, The abundance of sec-siRNAs that triggered by miR6027, miR482b and miR482e, respectively.

Figure S6


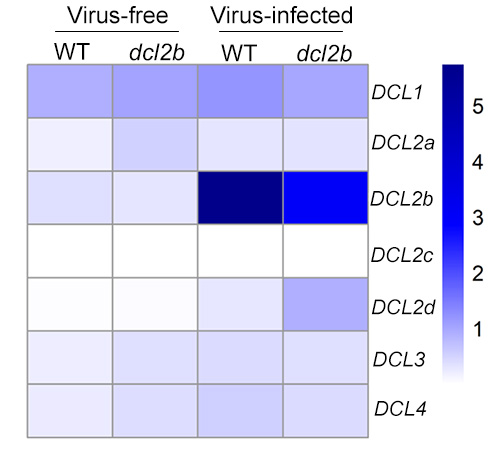


**Figure S6.** Heatmap of expression levels of seven *DCL* genes.
